# Digital resilience in Chinese adolescents: a portrayal of the current condition, influencing factors, and improvement strategies

**DOI:** 10.3389/fpsyt.2024.1278321

**Published:** 2024-02-29

**Authors:** Chunlin Qi, Nanchang Yang

**Affiliations:** Higher Institute of Teacher Education, Jiangxi Normal University, Nanchang, China

**Keywords:** adolescents, resilience, psychological, risk factors, coping strategies, digital technology

## Abstract

**Inroduction:**

Digital resilience is an important skill for adolescents in the digital age, but there is a lack of valid and reliable assessment methods. This study aimed to develop and validate a digital resilience questionnaire for Chinese adolescents based on the Digital Resilience Framework proposed by the UK Children’s Internet Safety Council

**Methods:**

This study employed a mixed research design, combining quantitative and qualitative data collected and analyzed. Over a six-month period (January to June 2023), a questionnaire was distributed to 12,208 adolescents from 10 high schools and 16 middle schools, with in-depth interviews performed with 10 of the participants.

**Results:**

The study revealed that Chinese adolescents digital resilience levels are slightly higher than average (M = 3.5038 > 3.5), but there is still potential for development, particularly in active learning. Additionally, a variety of characteristics influencing adolescents digital resilience were discovered, including gender, family residency, whether they are only children, grade level, the number of digital devices used per week, and the length of hours spent online daily.

**Discussion:**

This study developed and verified a digital resilience questionnaire for Chinese adolescents, which may be used to assess and improve their digital skills and well-being in the digital age. The study also identified various variables and themes that influence digital resilience, which can be used to navigate educational initiatives and policy. However, this study's shortcomings included a cross-sectional design, self-reported data, and cultural distinctiveness. Future research could address these limitations by undertaking a longitudinal study, utilizing numerous data sources, and contrasting different cultural contexts.

## 1 Introduction

Digital transformation has become an essential feature of contemporary social life and has profoundly impacted the field of education (1). As essential participants in the digital age, adolescents enjoy the conveniences and opportunities of digital technologies but also face many risks and challenges (2, 3). Improving adolescents’ digital resilience to effectively cope with and recover from unfavorable experiences in the digital space is an urgent issue. Digital resilience not only helps to protect adolescents’ mental health (2) but also helps to improve their academic performance (4) and social participation (5). This study adopts a mixed-methods design to collect data through questionnaires and interviews to analyze the current status of adolescents’ digital resilience and the relationship between personal characteristics, digital behaviors, and digital resilience as a means of exploring the level of digital resilience and its influencing factors among Chinese adolescents and to provide both theoretical and practical guidance for enhancing digital education for adolescents.

## 2 Literature review of digital resilience

The concept of resilience emerged in the disciplines of psychiatry and developmental psychology, owing to a growing interest in the individuality and developmental psychology of children who live in adverse circumstances (e.g., poverty, abuse, and neglect) but who can positively adapt and thrive in the face of such adversity (6). Resilience can be interpreted from two main perspectives: First, from the biological perspective, living organisms do not passively accept external forces and subsequently recover as physical materials do but have the instinct to adapt and self-adjust actively. Second, in the psychological sense, resilience not only implies that an individual can regain his or her initial state after significant trauma or stress, but it also implies that an individual can be resilient under the threat of stress. It focuses more on the individual’s growth and rebirth after a setback (7). In recent years, with the globalization of the international community and the continuous advancement of education reform, there have been many research systems with massive systems and a variety of perspectives on the theme of resilience, and more and more scholars are gradually expanding resilience to various fields, such as ecology, sociology, physics, and education. Given the digital revolution witnessed in this era, individuals are demonstrating new forms of resilience through technological innovation. This new type of resilience in the digital space is called “digital resilience.”

Because of the importance of digital technology in modern life, digital risks occur from time to time, and the concept and importance of digital resilience are attracting the attention of more and more researchers. Digital resilience is widely discussed as a concept but still needs a clear definition. Scholars have made numerous attempts to define digital resilience. Digital resilience is the use of technology to change practices to accommodate new environments while retaining the essential functions of the practice (8). Similarly, other researchers have defined digital resilience as “a dynamic personality attribute that comes from digital activation, whereby individuals activate digital resilience by experiencing and engaging in adequate digital risks and challenges online, rather than through avoidance and safety behaviors” (9).

Alternatively, digital resilience is “an evolving and dynamic process by which an individual or group learns how to identify, manage, and recover from online risks” (10). Building on these studies, Haiyan Sun et al. conceptualized individuals’ digital resilience based on the scope of schooling environments. They conceptualized the data through a conceptualization of digital resilience provided in 22 articles. Five key attributes of the conceptualization of digital resilience were extracted as follows: (1) Knowing online risks; (2) Being aware of solutions; (3) Acquiring knowledge and skills; (4) Bouncing back from stress; and (5) Continuing to move forward using self-efficacy (11). While there is no precise definition of digital resilience, many scholars regard it as a personal asset that promotes an individual’s continuing growth and development in the digital domain (12). Based on the preceding analysis, we believe that digital resilience is gradually formed by individuals living in the digital era while interacting with the digital space, which can encourage individuals to adapt to changes in the digital space, constantly take the initiative to carry out external interventions and self-intrinsic psychological regulation, and continuously build up the digital competency, psychological resilience, and quality of thinking required.

Reviewing the literature showed several areas for improvement in the research related to digital resilience. First of all, current research on digital resilience mainly focuses on children (13–15), elementary school students (14), college students (5, 8, 16–18), minority youth (19), consumers (20), and professionals (21), to name a few. In contrast, relatively little research has been conducted on adolescents in the middle and high school cohorts as a high online risk group. Second, while digital resilience is a fundamental skill for addressing digital risk issues, many articles focus solely on the level of resilience development of a specific risk issue, such as information leakage risk (21), technological risk (22), cyberbullying (23), and so on, resulting in insufficiently comprehensive research results. Furthermore, systematic reviews (24), conceptual models (11), and qualitative studies (4, 25) have dominated digital resilience research, and only a small minority of studies have attempted to use quantitative (5, 16, 26) or mixed research methods (22) to reveal the digital resilience of individuals. However, the role of demographic factors in influencing digital resilience remains to be clarified with specific analysis, and there is a need for in-depth research and an explanation of their relationship. Based on existing literature, only some empirical studies have explored Chinese teenagers’ digital resilience.

Furthermore, specific needs for more resources are scarce for assessing teenagers’ digital resilience. More studies on the subject of digital resilience in the adolescent population should be performed to help adolescents better adapt to digital life, as this is a critical issue in preparing responsible citizens who will not believe in fake news or be persuaded by extremist groups (27). An in-depth study of the development of digital resilience in adolescents and its influencing factors is a prerequisite and foundation for studying digital resilience in adolescents.

This study aims to investigate the digital resilience of teenagers (including middle and high school students) and identify the current state and individual influences on the development of digital resilience among Chinese adolescents. As a result, this research aims to create a digital resilience assessment framework for Chinese adolescents by combining the significant digital risks in the current network with the digital resilience development process framework established by the UKCIS Digital Resilience Working Group (9). Within this framework, the researchers designed a scale to explore the level of digital resilience among Chinese adolescents and its impact on adolescents’ digital resilience by gender, grade, home address, being an only child, the number of digital devices used per week, and the average number of hours spent on the Internet (including computers, cell phones, and other mobile devices), as well as to provide additional data and theoretical guidance for exploring and fostering digital resilience in adolescents.

With this goal in mind, the researchers endeavored to address the following research questions:

(1) What are the levels of digital resilience in adolescents regarding knowing the risks, seeking help, proactive learning, and self-recovery?
(2) What is the relationship between adolescents’ different genders, grades, home addresses, whether they are only children, the number of digital devices they use per week, the average number of hours they use the Internet, and digital resilience?

## 3 Materials and methods

### 3.1 Participants

To ensure that the sample was representative, the study adopted a stratified sampling method to collect it. The study sample comprised a variety of criteria, such as age, school type, gender, and geographical origin. The study specifically carried out the following steps: First, the participants were classified into a few age groups, such as 13–15 years old, 16–18 years old, 19–20 years old, and so on. Following that, participants were split into two groups based on the sort of school they attended, i.e., middle school and high school. The participants were subsequently separated into two groups based on their gender. Finally, individuals were referred to as living in urban or rural areas based on their geographic location. Random sampling was used inside each level to ensure that every student had an equal chance of being chosen. This was accomplished with the help of a random number generator, which avoided subjective bias. The study also selected the sample size to be sampled at every level. Finally, the samples from each stratum were combined to create a final sample set that confirmed the study’s representativeness. This stratified sample strategy contributed to an adequate representation of the different subgroups, making the study’s data more broadly relevant and reliable. Ultimately, the study covered 12,208 adolescents from the first year of middle school to the third year of high school. They ranged in age from 13 to 20 years. They came from 10 different Chinese cities and 26 schools (10 high schools and 16 middle schools). There were 6,034 from middle schools (49.4%) and 5,090 from high schools (50.6%). Regarding gender, there were 5,366 adolescent males (43.9%) and 5,758 female youths (56.1%). Regarding location, 4376 (35.8%) of adolescents settled in cities, while 6779 (61.2%) were in rural areas. This study’s respondents were selected from the 12,208 students who participated in it.

### 3.2 Instruments

The questionnaire adopts a Likert scale of 6, including personal data information and self-evaluation of Adolescent Digital Resilience.

#### 3.2.1 Personal data

Gender, grade level, family residence, whether or not the child was an only child, the number of times digital devices were used per week, and the average number of hours of internet use (including computers, cell phones, and other mobile devices) per day are among the six questions that were created.

#### 3.2.2 Adolescent digital resilience scale

The questionnaire collected data on adolescent digital resilience. The Adolescent Digital Resilience Questionnaire was adapted from the Digital Resilience Framework proposed by the UK Children’s Internet Safety Council. The Digital Resilience Framework consists of four elements: Understand (An individual understands when they are at risk online and can make informed decisions about the digital space they are in)、Know (An individual knows what to do to seek help from a range of appropriate sources)、Learn (An individual learns from their experiences and can adapt their future choices, where possible) and Recover (An individual can recover when things go wrong online by receiving the appropriate level of support to aid recovery) (9). The study appropriately adopted the digital resilience framework’s four components to form the Digital Resilience Questionnaire for Adolescents: Knowing the risks, Seeking help, Proactive learning, and Self-recovery (see **Table 1**).

**Table 1:** Dimensions of adolescent digital resilience scale.

Dimension	Item distribution	α	Example
1. Knowing the risks	7, 8, 9	0.862	I understand the various online risks.
2. Seeking help	10,11,12,13,14	0.912	When I was in danger online, I learned how to seek effective offline assistance.
3. Proactive learning	15,16,17	0.858	I can learn strategies for dealing with risk from the online risks I’ve encountered in the past.
4. Self-recovery	18,19	0.808	When I encounter cyber risks, I can recover a good mental state in time.
Total	─	0.964	**─**

These four dimensions are described in detail below:

#### 3.2.3 Knowing the risks

This component demonstrates adolescents’ power to recognize and respond to online threats. Adolescents who understand risks can identify and circumvent online threats such as cyberbullying, pornography, false information, and online fraud; protect their personal information and privacy; do not trust strangers or online advertisements; do not download or click on suspicious links or files; use the Internet wisely; do not become overly obsessed with online games or social media; and do not allow adverse factors to affect their mental health They will not allow abusive remarks or pressure from the Internet to have an impact on their mental health (28).

#### 3.2.4 Seeking help

When adolescents experience internet challenges, this aspect illustrates their willingness and ability to seek help. Adolescents who can actively seek help can take the initiative to seek assistance from trusted individuals or organizations such as parents, teachers, friends, police officers, cybersecurity experts, and so on, as well as use online resources and tools to solve online problems they encounter on their own (29).

#### 3.2.5 Proactive learning

This factor represents adolescents’ capacity to learn and grow from internet encounters. Adolescents with active learning abilities may reflect on their online behaviors and consequences, learn from their mistakes, and summarize their successes (30); they may additionally actively explore and try out new online skills and knowledge, such as programming, designing, creating, and researching; and they might formulate and implement reasonable online learning plans and strategies based on their interests and goals.

#### 3.2.6 Self-recovery

This variable suggests adolescents’ ability to recover and adjust after experiencing abuse online. Adolescents with self-recovery capabilities may implement practical steps to lessen and eradicate the impacts of online victimization, such as eliminating or reporting inappropriate content, changing or resetting passwords, disconnecting or replacing network devices, and so on. They may also modify their thinking and emotions, meet online problems and difficulties with positive and hopeful attitudes, and rely on the assistance and support of others to regain self-confidence and self-esteem and reintegrate into the world of the Internet (31). They can also use the help and encouragement of others to reestablish their self-esteem and confidence and reintegrate into the online social and learning environment.

The questionnaire’s four dimensions comprise 13 six-point Likert scale questions ranging from 1 (strongly disagree) to 6 (strongly agree). Higher ratings exhibit the adolescent’s digital resilience.

The interviews intended to supplement the quantitative analysis results by further exploring and understanding the current state of teenagers’ digital resilience and the role of adolescents’ individual characteristics on digital resilience. Through qualitative data, we could probe individual-specific learning experiences of digital resilience and discuss the issues in depth and detail.

The interviewees were selected from among the students who provided contact information in the questionnaire. A total of 10 students volunteered for the interview.

#### 3.2.7 Interview participants

After analyzing the quantitative data, the researchers identified adolescents with low and high digital resilience scores. Through purposeful sampling, 10 participants agreed to participate in the interview. Before data collection, researchers obtained participants’ consent by ensuring the anonymity, privacy, and confidentiality of participant information.

The interview questions are all related to digital resilience in adolescents, especially how adolescents can quickly and seamlessly adopt new digital technology solutions to recover, bounce back, and move forward if things go wrong in the digital environment (11).

The interviews were conducted in Chinese, so the students could understand the questions and express their opinions freely and clearly.

### 3.3 Procedure

This study adopted a mixed research methodology, combining quantitative and qualitative data collection and analysis.

Firstly, this study was based on quantitative research with an extensive questionnaire survey of Chinese adolescents (middle school and high school students) from January 2023 to June 2023, covering 10 high schools and 16 middle schools with 12,208 participants. The questionnaires were distributed via email during this phase of the research, ensuring the participants’ anonymity and the data’s confidentiality. To verify the questionnaire’s validity, we referred to relevant research findings on digital resilience and conducted a small-scale test during the design process. Moreover, to acquire a deeper understanding of teenagers’ perspectives, we modified the questionnaire while conducting interviews to ensure that it could accurately and effectively gather adolescents’ experiences regarding digital resilience.

Secondly, in the qualitative phase of the investigation, in-depth individual interviews were carried out with 10 of the 12,208 adolescents who submitted the questionnaire. This round of interviews aimed to obtain information about how adolescents deal with certain digital potential risks and attain digital resilience in their daily lives. To provide flexibility while covering crucial concerns, the interviews were done in a semi-structured style. We produced a bespoke interview guide based on the answers to the questionnaire to lead the discussion on the issue of external support and personal digital resilience factors. During the interviews, we considered the young people’s explanations of external support and personal digital resilience factors and how these interacted in their particular situations to create excellent digital resilience results. The interview questions were meant to be open-ended so that they could be easily altered in response to the participant’s responses, allowing us to dig deeper into the significant issues and collect rich data regarding adolescents’ everyday activities. Questions ranged from the influence of significant others to their talents, shortcomings, interests, and life events, with the goal of gaining a broad picture of adolescents’ opinions and experiences. Each interview was audio recorded and verbatim transcribed. In addition, the researcher made field notes during the interviews and gave more specific descriptions to understand the participants’ comments better. We aimed to synthesize quantitative and qualitative data to examine adolescents’ digital resilience and its affecting aspects comprehensively and in-depth, employing this mixed research approach.

### 3.4 Data collection and analysis

First, this study utilized a questionnaire survey method to obtain quantitative data on the current status of digital resilience among Chinese adolescents. A total of 12,208 questionnaires were collected, covering 10 high schools and 16 middle schools. To ensure the quality and validity of the data, the researchers cleaned and checked the obtained questionnaires. Invalid answers, including answers that did not meet the requirements of the questions, apparent contradictions or logical errors with other answers to the questions, and traces of intentional or random filling in, were eliminated, and a total of 1,083 were eliminated. Eventually, 11,125 satisfactory answers were left for further analysis and discussion.

In the quantitative analysis stage of the questionnaire data, the researchers used SPSS 26.0 statistical software. The internal consistency and reliability of the questionnaire were examined, and the results showed that the scale had good reliability (α =.964). The KMO coefficient of the scale was.986, and the significance of Bartlett’s test of sphericity was less than 0.001 (p<.001), indicating that the questionnaire’s structural validity was good. To validate the structural model of the questionnaire further, the researchers used AMOS 24 software for confirmatory factor analysis (CFA). Use the below goodness-of-fit indices: chi-square statistics; χ2/df ratio, comparative fit index (CFI), goodness-of-fit index (GFI), Tucker-Lewis index (TLI), and the Mean Square Error of Approximation (RMSEA). The model is considered an acceptable fit if χ2/df < 3. Values higher than 0.90 denote an acceptable model fit for CFI, GFI, and TLI.

For RMSEA, values lower than 0.08 represent a good fit (32). The results showed that the model fit performed well on all indices and met the acceptance criteria. Furthermore, the researchers employed Pearson correlation coefficients to analyze the associations between digital resilience and various individual characteristics. The correlation coefficient expresses the strength of the relationship. If the absolute value of the correlation coefficient is less than or equal to.39, it manifests a low correlation. It reflects the degree of the correlation. If the absolute value of the correlation coefficient is between.40 and.69, it shows a moderate correlation. If the absolute value of the correlation coefficient is above.70 and 1, it demonstrates a high correlation (33). T-tests were utilized to explore disparities in the four factors of the Adolescent Digital Resilience Scale based on gender, family residence, and whether an individual is an only child. Meanwhile, the chi-square test was applied to assess distinctions in the four factors of digital resilience concerning grade level, number of digital devices used per week, and length of Internet surfing every day.

Second, in the qualitative part of the study, individual semi-structured interviews were used to gain insight into the adolescents’ perspectives and experiences. By coding and analyzing the data obtained from interviews and observations using NVIVO 11 software, we ensured a systematic organization of the qualitative data. Interviews were audio-recorded, transcribed, and translated from Chinese to English to better understand participants’ responses. All digitized, usable notes from the survey period were also integrated into the study after the participants made revisions. Each transcript was individually analyzed to ensure a thorough understanding of the qualitative data. All participants’ names were anonymized throughout the study to ensure privacy and confidentiality.

## 4 Results

### 4.1 Result1: CFA

**Figure 1:**
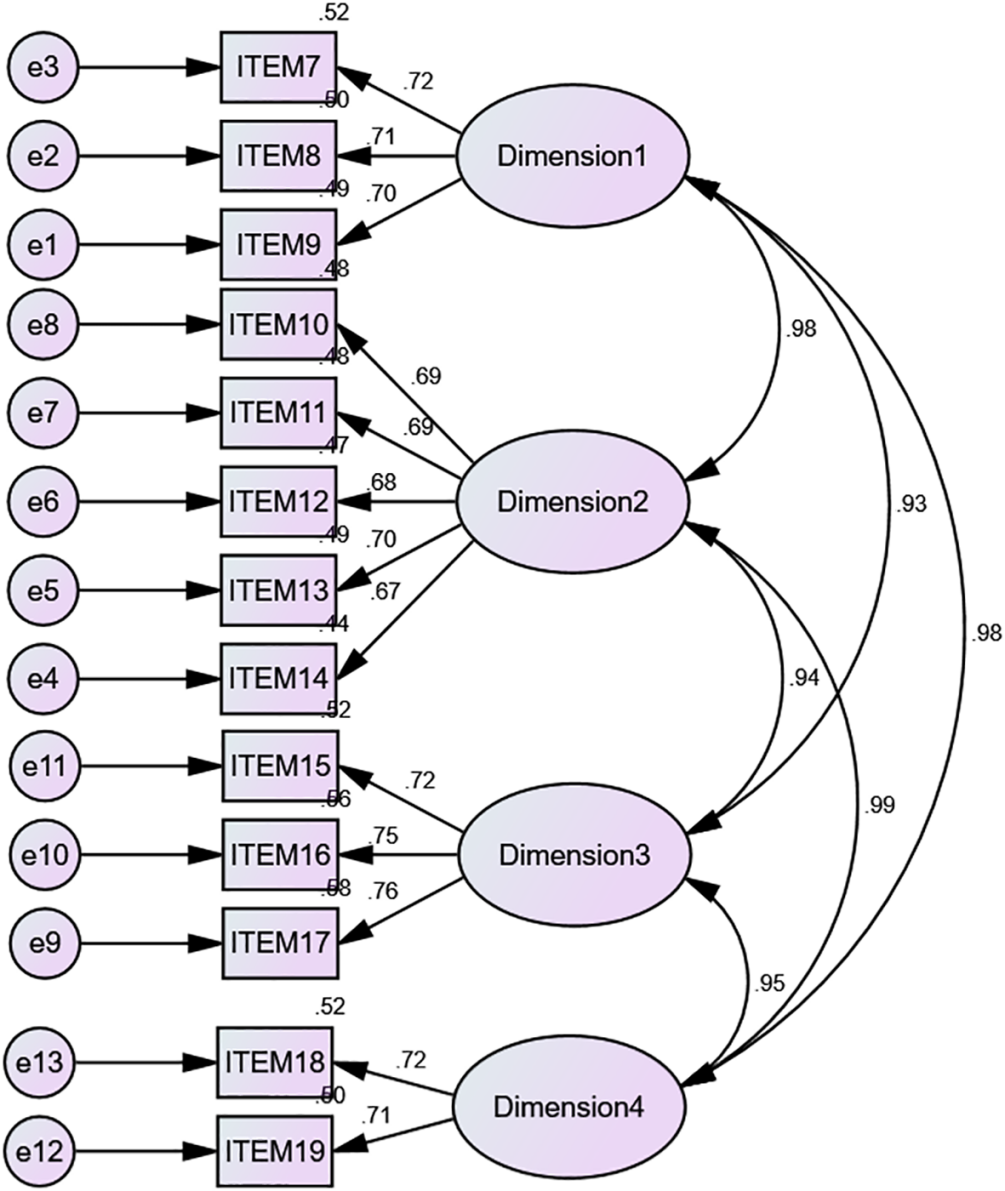
Diagram of a measurement model of digital resilience in adolescents.

The measurement model of adolescent digital resilience consists of four latent factors (knowing the risks, Seeking help, Proactive learning, and Self-recovery) and 13 observed variables. The CFA of the measurement model provided a good fit to the data. χ ^2^ = 77.408, d f =59, p < 0.001, χ ^2^/d f =1.312, RMSEA = .026, CFI = .994, GFI = .975, TLI = .991 (**Figure 1**).

### 4.2 Result2: Descriptive statistics of digital resilience in adolescents

A descriptive analysis was used to examine the levels of adolescents’ Digital resilience. The analysis results are shown in **Table 2**.

**Table 2:** General descriptions of digital resilience in adolescents (N=11125).

Item	Min	Max	M	SD
Knowing the risks	1	6	3.5047	1.42135
Seeking help	1	6	3.5047	1.37352
Proactive learning	1	6	3.4999	1.40478
Self-recovery	1	6	3.5058	1.45708
Digital resilience	1.28	5.92	3.5038	1.33676

A 6-point Likert scale was used to measure Digital Resilience for adolescents, with a median of 3.5. Higher scores Suggest higher levels of Digital Resilience. As displayed in **Table 2**, the mean score of the Digital Resilience in adolescents is higher than the median, which indicates that the Digital Resilience in adolescents is above the mid-point of a scale of six (M=3.5038; SD=1.33676). Among the four factors of Digital Resilience for adolescents, the mean of self-recovery is the highest (M=3.5058; SD= 1.4568). The mean of Knowing the risks (M= 3.5047; SD =1.42135) and Seeking help (M= 3.5047; SD =1.37352) is tied for second place. The mean of Proactive learning is the lowest (M=3.4999; SD = 1.40478), which indicates that adolescents in China have a moderate and low level of Proactive learning ability.

### 4.3 Result3: Digital resilience analysis of different individual characteristics of adolescents in China

#### 4.3.1 Correlation analysis of digital resilience and different individual characteristics

As shown in **Table 3**, the correlation coefficient between various individual characteristics of adolescents and digital resilience with gender, grade level, family residence, academic segments, and number of times using digital devices per week correlates moderately with knowledge of risks, seeking help, proactive learning, self-help, and digital resilience. Furthermore, the average number of hours of internet use (including computers, mobile phones, and other mobile devices) per day correlates highly with risk knowledge, help-seeking, proactive learning, self-recovery, and digital resilience. In addition, whether or not the child was an only child correlated modestly with risk knowledge, help-seeking, proactive learning, self-recovery, and digital resilience.

**Table 3:** The correlation between different individual characteristics and digital resilience in general.

Item	Knowing the risks	Seeking help	Proactive learning	Self-recovery	Digital Resilience
Gender	.620^**^	.638^**^	.614^**^	.591^**^	.651^**^
Grade level	.591^**^	.604^**^	.585^**^	.567^**^	.620^**^
Family residence	.589^**^	.597^**^	.578^**^	.566^**^	.616^**^
Only-child or non-only-child	.266^**^	.272^**^	.259^**^	.256^**^	.278^**^
The number of times digital devices were used per week	.639^**^	.652^**^	.632^**^	.609^**^	.669^**^
The average number of hours of internet use per day	.726^**^	.746^**^	.722^**^	.701^**^	.765^**^

^**^Correlation is significant at the 0.01 level (two-tailed).

#### 4.3.2 Gender dimension

Proactive learning is generally low, and there are gender differences in Knowing the risks, Seeking help, and Self-recovery. The analysis results are presented in **Table 4**.

**Table 4:** Mean scores, standard deviations, and gender differences in means for the four digital resilience items.

Item (N=11125)	Girl (N=6758)		Boy (N=4366)		Gender difference	
	M	SD	M	SD	Cohen’s d	T
Knowing the risks	2.7958	1.05339	4.6018	1.20364	1.58224	83.436^***^
Seeking help	4.5942	1.15404	2.8008	.99142	1.58210	87.282^***^
Proactive learning	2.8066	1.04241	2.5733	1.00426	1.55603	82.061^***^
Self-recovery	3.5768	1.25742	2.8139	1.11969	.326660	17.227^***^
Digital resilience	2.8043	.942270	4.5866	1.11769	1.71527	90.455***

*** represents p < 0.001, demonstrating that the results are highly significant at the 0.1% level of significance.

T-tests were conducted to examine gender differences for the four factors of the Adolescent Digital Resilience Scale. Results from the Independent Samples T-test (p<.001) showed that, in terms of gender, boys’ digital resilience was significantly higher than girls’ digital resilience. Proactive learning scores were generally low, but when analyzed across the four dimensions of digital resilience, the differences were significant in Knowing the risks (p<.001), Proactive learning (p<.001), and Self-recovery (p<.01). This suggests that adolescents are less willing to learn about knowledge of digital risks actively and that female adolescents are better than male adolescents at Seeking help, proactive learning, and Self-recovery.

In addition, as displayed in **Table 4**, gender differences for all four factors were found: Girls scored significantly higher on Seeking help, Proactive learning, and Self-recovery, whereas Boys scored higher on Knowing the risks. Moreover, the values of Cohen’s d indicated that the gender differences were significant for all factors other than Self-recovery, where a big-sized gender difference emerged whereby females had, on average, a substantially higher score than males.

#### 4.3.3 Family residence dimension

The difference in self-recovery was insignificant for rural students and was significantly lower than for urban students in the remaining aspects. The analysis results are presented in **Table 5**.

**Table 5:** Mean scores, standard deviations, and family residence differences in means for the four digital resilience items.

Item (N=11125)	urban (N= 4346)		rural (N= 6779)		Gender difference	
	M	SD	M	SD	Cohen’s d	T
Knowing the risks	4.5504	1.28071	2.8343	1.05525	1.45786	76.884^***^
Seeking help	4.5280	1.24047	2.8486	1.00394	1.48649	78.394^***^
Proactive learning	4.5146	1.28682	2.8494	1.04583	.469820	24.777^***^
Self-recovery	4.5361	1.31761	2.8452	1.11996	.272810	14.386
Digital resilience	4.5323	1.19977	2.8444	.946990	1.56425	82.491^***^

*** represents p < 0.001, demonstrating that the results are highly significant at the 0.1% level of significance.

The results of the Independent Samples T-test (t=82.491, p<.001) showed that the digital resilience of urban adolescents (M=4.5323, SD=1.19977) was significantly higher than that of rural adolescents (M=2.8444, SD=.94699) in terms of where the adolescents’ families lived. Analyzed the four dimensions of digital resilience, As displayed in Table, in terms of Knowing the risks (t=76.884, p<.001), seeking help (t=78.394, p=.000<.001), and Proactive learning (t=24.777, p<.001), the difference is significant. Specifically, urban adolescents are significantly better than rural adolescents in knowing risks, seeking help, and actively learning. There was no significant difference in the self-recovery dimension (t=14.386, p =.418>.05). And, the values of Cohen’s d indicated that the family residence differences were significant for all factors other than Self-recovery and Proactive learning, where a significant gender difference emerged whereby urban students had on average a substantially higher score than rural students.

#### 4.3.4 Only-child dimension

The digital resilience of non-only-child adolescents is significantly higher than that of only-child adolescents. The results of the analysis are presented in **Table 6**.

**Table 6:** Mean scores, standard deviations, and one-child differences in means for the four digital resilience items.

Item (N=11125)	Non-only child (N= 5120)		Only-child (N= 6005)		Only-child difference	
	M	SD	M	SD	Cohen’s d	T
Knowing the risks	4.4890	1.23164	2.6655	.95870	1.66335	87.721^***^
Seeking help	4.4744	1.18212	2.6779	.90462	1.5372	90.675^***^
Proactive learning	4.4608	1.23404	2.6807	.94826	.11233	5.924^***^
Self-recovery	4.4674	1.27358	2.6858	1.04389	1.5372	81.068^***^
Digital resilience	4.4729	1.14855	2.6775	.83799	1.80234	-95.047^***^

^***^Correlations are significant at the 0.001 level (two-tailed).

The results of the Independent Samples T-test (t=-95.047, p<.001) showed that the numerical resilience of adolescents with non-only children (M=4.4729, SD=1.14855) was significantly higher than that of adolescents with only children (M=2.6775, SD=.83799) in terms of being an only child. When analyzed across the four dimensions of digital resilience, adolescents with non-only children also scored higher than adolescents with only children on the dimensions of Knowing the risks (p<.001), Seeking help (p<.001), Proactive learning (p<.001) and Self-recovery (p<.001). Moreover, the values of Cohen’s d indicated that the difference between an only child and a non-only child was significant for all factors other than Proactive learning, where a big-sized difference emerged whereby students from one-child families had a substantially higher score than students from one-child families.

#### 4.3.5 Grade level dimension

The higher the grade level, the higher the digital resilience, with little overall difference for junior high school students and the highest level of digital resilience for High School Year 2. The analysis results are presented in **Table 7**.

**Table 7:** Mean scores, standard deviations, and grade level differences in means for the four digital resilience items.

Item (N=11125)	Grade level	df	M	SD	F	P	Partial η ^2^
Knowing the risks	Junior high school 1	1934	2.7099	.95883	2301.992^***^	.000	.509
	Junior high school 2	2094	2.7688	1.05165			
	Junior high school 3	2003	2.6717	.96604			
	High School Year 1	1616	3.37	1.26714			
	High School Year 2	2691	5.1493	.76073			
	High School Year 3	781	4.1948	1.08953			
Seeking help	Junior high school 1	1934	2.7102	.90645	2535.655^***^	.000	.533
	Junior high school 2	2094	2.7843	.99484			
	Junior high school 3	2003	2.6807	.89708			
	High School Year 1	1616	3.3792	1.21015			
	High School Year 2	2691	5.1343	.69825			
	High School Year 3	781	4.1611	1.04541			
Proactive learning	Junior high school 1	1934	2.7299	.95309	2180.707^***^	.000	.495
	Junior high school 2	2094	2.7569	1.03082			
	Junior high school 3	2003	2.7063	.95351			
	High School Year 1	1616	3.3704	1.27076			
	High School Year 2	2691	5.1023	.80356			
	High School Year 3	781	4.1816	1.09318			
Self-recovery	Junior high school 1	1934	2.7207	1.04853	1951.434^***^	.000	.467
	Junior high school 2	2094	2.7792	1.11668			
	Junior high school 3	2003	2.6939	1.03108			
	High School Year 1	1616	3.3745	1.31655			
	High School Year 2	2691	5.122	.81858			
	High School Year 3	781	4.1829	1.19101			
Digital resilience	Junior high school 1	1934	2.7177	.84369	2828.247^***^	.000	.560
	Junior high school 2	2094	2.7723	.93511			
	Junior high school 3	2003	2.6881	.83584			
	High School Year 1	1616	3.3735	1.1691			
	High School Year 2	2691	5.127	.66312			
	High School Year 3	781	4.1801	.98037			

*** represents p < 0.001, demonstrating that the results are highly significant at the 0.1% level of significance. Item (N = 11125) means the total sample size for each item, which is 11125 adolescent students. Grade level means the different grades, which are six in total, namely Junior High School Year 1, Junior High School Year 2, Junior High School Year 3, Senior High School Year 1, Senior High School Year 2, and Senior High School Year 3. df means the degrees of freedom, which is the number of data points that can vary freely when calculating the statistic. M means the mean, which is the average score for each grade on each item. SD means the standard deviation, which is the degree of dispersion of the scores for each grade on each item. F means the F-test statistic, which is the indicator used to test whether the means of different grades have significant differences. P means the significance level of the F-test, which is the probability of rejecting the null hypothesis. Partial η2 means the effect size, which is the proportion of the total variation explained by the mean difference between different grades.

The chi-square test showed significant differences in digital resilience (p<.01) between the adolescents. There are significant differences in all dimensions. Specifically, there were significant differences in four dimensions: Knowing the risks (p<.001), Seeking help (p<.001), Proactive learning (p<.001), and Self-recovery (p<.001). Among them, High school sophomores scored the highest (M=5.127, SD=.66312), followed by High School Year 3 (M=4.1784, SD=.9799) and High School Year 1 (M=3.3735, SD=1.1691). There is little difference among junior high school students. On the other four dimensions of digital resilience, students in their second year of high school continue to perform best. Moreover, the values of Partial η 2 indicated that the difference between grade levels was significant for all factors, where a big-sized difference emerged whereby High school students had, on average, a substantially higher score than junior high school students.

#### 4.3.6 Dimension of number of digital devices used per week

Digital resilience is highest among adolescents who use digital devices 4-6 times weekly. The analysis results are presented in **Table 8**.

**Table 8:** Mean scores, standard deviations, and number of digital devices used per week: differences in means for the four digital resilience items.

Item (N=11125)	Number of times	df	M	SD	F	P	Partial η ^2^
Knowing the risks	0 times	2350	2.5486	.89564	4104.683^***^	.000	.596
	1-3 times	2296	2.5464	.90589			
	4-6 times	2945	5.2525	.70696			
	7-9 times	1762	3.2893	1.03995			
	10 times or more	1767	3.3237	1.04449			
Seeking help	0 times	2350	2.5494	.83249	4679.541^***^	.000	.627
	1-3 times	2296	2.5688	.84448			
	4-6 times	2945	5.2391	.63765			
	7-9 times	1762	3.2842	.97244			
	10 times or more	1767	3.3206	.97838			
Proactive learning	0 times	2350	2.5657	.89718	4021.032^***^	.000	.591
	1-3 times	2296	2.5639	.89363			
	4-6 times	2945	5.2245	.71082			
	7-9 times	1762	3.2823	1.02167			
	10 times or more	1767	3.3017	1.04264			
Self-recovery	0 times	2350	2.5625	.97671	3341.447^***^	.000	.546
	1-3 times	2296	2.5725	.99513			
	4-6 times	2945	5.2203	.78365			
	7-9 times	1762	3.2754	1.11627			
	10 times or more	1767	3.3453	1.11732			
Digital resilience	0 times	2350	2.5565	.76891	5382.012^***^	.000	.659
	1-3 times	2296	2.5629	.77618			
	4-6 times	2945	5.2341	.59831			
	7-9 times	1762	3.2828	.90632			
	10 times or more	1767	3.3228	.92019			

*** represents p < 0.001, demonstrating that the results are highly significant at the 0.1% level of significance. Item (N = 11125) means the total sample size for each item, which is 11125 adolescent students. The number of times means the number of times using digital devices per week, which has five categories: 0 times, 1-3 times, 4-6 times, 7-9 times, and 10 times or more. df means the degrees of freedom, which is the number of data points that can vary freely when calculating the statistic. M means the mean, which is the average score for each category on each item. SD means the standard deviation, which is the degree of dispersion of the scores for each category on each item. F means the F-test statistic, which is the indicator used to test whether the means of different categories have significant differences. P means the significance level of the F-test, which is the probability of rejecting the null hypothesis. Partial η2 means the effect size, which is the proportion of the total variation explained by the mean difference between different categories.

The research shows that adolescents who use digital devices 4-6 times a week have the highest digital resilience score, while adolescents who use digital devices 0 times a week have the lowest digital resilience score. Specifically, Specifically, 4-6 times (M=5.2341, SD=.59831) > 10 times or more (M=3.3228, SD=.92019) > 7-9 times (M=3.2828, SD=.92019) > 1-3 times (M=2.5629, SD=.77618) > 0 times (M=2.5565, SD= .76891). Furthermore, adolescents who use digital devices 4-6 times per week score higher and perform better on the four dimensions of digital resilience.

#### 4.3.7 Dimension of the length of surfing the Internet every day

Adolescents who use 1-2 hours daily have the highest digital resilience. The analysis results are presented in **Table 9**.

**Table 9:** Mean scores and standard deviations and the length of Internet surfing every day differ in means for the four digital resilience items.

Item (N=11125)	length of time	df	M	SD	F	P	Partial η ^2^
Knowing the risks	0-0.5 hours	2300	2.5279	0.87281	4567.035^***^	.000	.622
	0.5-1 hour	2306	2.5232	0.86808			
	1-2 hours	3035	5.2602	0.69104			
	2-3 hours	1692	3.2388	0.98711			
	3 hours or more	1787	3.2992	1.03535			
Seeking help	0-0.5 hours	2300	2.5472	0.81364	5311.693^***^	.000	.656
	0.5-1 hour	2306	2.5316	0.80926			
	1-2 hours	3035	5.2518	0.62053			
	2-3 hours	1692	3.222	0.87828			
	3 hours or more	1787	3.2935	0.97633			
Proactive learning	0-0.5 hours	2300	2.543	0.87319	4400.92^***^	.000	.613
	0.5-1 hour	2306	2.5623	0.87374			
	1-2 hours	3035	5.2301	0.71384			
	2-3 hours	1692	3.2128	0.97259			
	3 hours or more	1787	3.2752	1.01397			
Self-recovery	0-0.5 hours	2300	2.5582	0.95808	3648.331^***^	.000	.568
	0.5-1 hour	2306	2.544	0.96934			
	1-2 hours	3035	5.2279	0.78417			
	2-3 hours	1692	3.2442	1.03650			
	3 hours or more	1787	3.2894	1.12246			
Digital resilience	0-0.5 hours	2300	2.5441	0.73985	6091.922^***^	.000	.687
	0.5-1 hour	2306	2.5403	0.74334			
	1-2 hours	3035	5.2425	0.59731			
	2-3 hours	1692	3.2295	0.82439			
	3 hours or more	1787	3.2893	0.90474			

*** represents p < 0.001, demonstrating that the results are highly significant at the 0.1% level of significance. Item (N = 11125) means the total sample size for each item, which is 11125 adolescent students. length of time means the length of Internet surfing every day, which has five categories: 0-0.5 hours, 0.5-1 hour, 1-2 hours, 2-3 hours, and 3 hours or more. df means the degrees of freedom, which is the number of data points that can vary freely when calculating the statistic. M means the mean, which is the average score for each category on each item. SD means the standard deviation, which is the degree of dispersion of the scores for each category on each item. F means the F-test statistic, which is the indicator used to test whether the means of different categories have significant differences. P means the significance level of the F-test, which is the probability of rejecting the null hypothesis. Partial η2 means the effect size, which is the proportion of the total variation explained by the mean difference between different categories.

Through Chi-square test analysis, it is concluded that there are significant differences in digital resilience among adolescents who use the Internet at different lengths (p<.001). There are significant differences in four dimensions: Knowing the risk (p<.001), Seeking help (p<.001), Proactive learning (p<.001), and Self-recovery (p<.001). Adolescents who use the Internet for 1-2 hours a day have the highest digital resilience scores, and adolescents who use digital devices for 0.5–1 hour a day have the lowest digital resilience scores. Specifically, 1-2 hours (M=5.2425, SD=.59731)>3 hours or more (M=3.2893, SD=.90474)>2–3 hours (M=3.2295, SD=.82439)>0.5–1 hour (M=3.2295, SD=.82439)>0.5–1 hour (M=2.5403, SD=.74334)>0-0.5 hour (M=2.5441, SD=.73985) Moreover, adolescents who use digital devices for 1-2 hours a day score higher and perform better on the four dimensions of digital resilience.

## 5 Discussion

### 5.1 General descriptions of digital resilience of adolescents

The descriptive results show that digital resilience in adolescents is above the midpoint of a scale of six. The adolescents have good digital resilience, enabling them to cope with and deal with digital risks and challenges in the online environment. This means that students can understand the potential risks in online learning, better control their emotions, proactively self-regulate, seek external support, build interpersonal support, and deal with risks in online learning with a positive mindset.

These results are similar to those of Ragni et al. (16), which indicated that Italian higher education students had a high level of digital resilience. These data are in line with previous studies from other countries. For example, Eri and colleagues (5) found that university students in Australia and Asia showed good digital resilience during the 2019 coronavirus disease epidemic. These results indicate that students have good digital resilience, whether they are middle school, high school, or college students.

Self-recovery is the first important factor affecting adolescent digital resilience. Eri et al. (5) claimed that digital resilience is the psychological ability of individuals to maintain function by recovering from, adapting to, and learning from adversity generated by the use of digital technologies in higher education settings. Digital recovery is integral to an individual’s digital development when adopting new digital technologies. Individuals can adapt quickly as they adjust to the digital threats and risks they face based on the situation. Similarly, Ragni et al. (16) found that among students with high levels of digital resilience, about 42 percent admitted that they recovered quickly all or most of the time after facing difficulties with digital technology, and another 45 percent of respondents noted that they recovered quickly some of the time. Thus, recovery represents a person bouncing back to their normal activities, just as before the stressful event occurred (11). Self-recovery is an essential factor in developing an individual’s digital resilience. This result is also reflected in the interviews in this study. For example, When asked how soon you can recover physically and mentally when exposed to online risks, Student D stated, *“I feel that when I encounter an online risk, I can quickly calm down and then quickly return to my normal mental state.”* Student K said, *“When I get cyber-attacked online, I feel bad at first. However, I can get myself out of this distress as soon as possible.”.* This confirms the quantitative results showing that adolescents can quickly recover an excellent physical and mental state and maintain a high level of digital resilience when exposed to digital risks.

The most critical and essential aspects of digital resilience in adolescents are knowing the risks and seeking help. According to the research, the understanding risk was the first word used in the digital resilience field. The study by Sun et al. (11) concluded that Individuals recognize risks or threats online and can make informed decisions about the digital spaces in which they live. Students perceive different risks or threats in online learning, such as problematic online behaviors, cyberbullying, and failed webinars. Therefore, knowing the risks is a prerequisite for developing digital resilience in adolescents. Similarly, Sharma et al. (2) found that digitally resilient students report that they can recognize risks or threats online and make informed decisions about the digital environment in which they live. Moreover, Hammond et al. (34) concluded that in a risk society, the ability to identify risk means that the same risk is something that individuals can manage and prevent. Therefore, the digital resilience of individuals is possible when digital risks are identified and controlled. In the interviews, most students believed knowing the risks was the first step in developing digital resilience. For instance, Student A believes recognizing online risks is a prerequisite for avoiding and dealing with them. Student H believes that the harm from online risks is not direct. However, identifying these risks enables people to leverage the advantages of the internet better.

Secondly, the results of the current mixed-methods study also revealed that help-seeking is an essential influencer of digital resilience in adolescents. The results of the present study are in line with Eri et al. (5) in that they pointed out that when college students encounter difficulties while studying online, most of them seek outside help. The survey results show that 66% of participants sought self-help, and 27% received help from peers. External resources and other sources shared the remaining 7%. The results of the present study also sit well with Tian et al. (35) in that external support is a great support system to promote resilience in adolescents.

Despite the differences among participants, seeking support is an essential factor influencing adolescents’ digital resilience in the current study. Seeking external support provides strong support for adolescents facing online risks. Interviews also confirm this result. Of the 10 respondents, 7 said they would seek help from their parents, teachers, or classmates if they had trouble online. However, the type of help varies from person to person. At the same time, respondents said they are happy to help others and face online risks together. For example, Student C stated that we are not supermen and cannot solve all the problems we encounter on our own, so it is essential to seek help when we encounter risks while surfing the Internet.

Most interviewees emphasized the importance of seeking help during cyber-risk exposure. Faced with diverse pressures and challenges during the online period, external support is sought from various sources, including parents, relatives, friends, teachers, organizations, and social media platforms. External support is one of the key factors contributing to the development of psychological resilience in adolescents. It contributes to a sense of security, belonging to family and school, social inclusion, participation, love, recognition of one’s social status, and freedom from discrimination. Adolescents’ social networks can contribute to forming their digital resilience through the practical and emotional resources generated by these connections.

Proactive learning is a crucial factor influencing the digital resilience of adolescents. Quintiliani et al. (36) reported that active learning of experiences and ways to solve difficulties from bad experiences is a protective factor in developing individual resilience. Studies are parallel to this. Ragni et al. (16) found that learning knowledge and skills from negative online experiences can help them better cope with future risks encountered in cyberspace. According to the literature, these are all the main attributes of a good level of digital resilience. According to the literature, active learning is a crucial attribute of individuals with good levels of resilience and digital resilience. However, the results showed that students performed poorly in active learning. However, the importance of active learning must be addressed. In interviews, ten students said they had little time to learn about cybersecurity and risks. This finding also indicates that schools and teachers attach little importance to cyber security awareness education for adolescents.

### 5.2 The Correlation between adolescent digital resilience and different individual characteristics

The study’s correlational analysis revealed a high positive correlation between adolescents’ digital resilience and personal characteristics, including gender, grade level, family residence, the number of times digital devices were used per week, and the average number of hours of internet use per day. It shows a strong correlation between the influence of individual characteristics on adolescents’ digital resilience. The results of the present study align with Budak et al. (20) in that they pointed out that the demographic characteristics of individuals are an essential influence on resilience in online environments. The results of the present study also sit well with the fact that the most essential prerequisites for personal resilience include various psychological factors (37). In contrast, other factors fall into sociodemographic categories, typically including income, educational attainment, age, occupation, and age (38).

Adolescents’ average daily time online plays the most influential role in adolescent digital resilience. The study showed that adolescents who used technology for 1-2 hours daily had the highest digital resilience. According to the study, spending 1-2 hours a day online is most beneficial for teenagers. Shortening time on the Internet is not conducive to young people enjoying the benefits of the Internet for learning, entertainment, and communication. Too much time on the Internet can lead to threats to the rights, safety, and mental health of youth from online sexual assault, harmful content, false information, and cyberbullying. A moderate amount of time spent online is suitable for young people to enjoy the benefits of the Internet and develop their digital resilience. However, it also contributes to their physical and mental health. Data analysis showed that teens who use digital devices 4-6 times per week are the most digitally resilient. Research has demonstrated that the time individuals spend online positively correlates with their ability to adapt to online privacy violations. The present mixed-methods study reveals that teenagers should not spend less time online each day; spending more time online each week is better. This data analysis shows that 1-2 hours of Internet access per day and 4-6 times per week are most beneficial to developing digital resilience in adolescents.

Gender shows a positive correlation with adolescent digital resilience. The present study suggests that boys perform better than girls in digital resilience. Similarly, Zhang et al. (39) found that men are more interested in digital technology, more digitally savvy, more likely to take active control, and more willing to take risks than women. As such, the OECD (40) has identified the existence of the so-called “digital gender divide.” Generally, men have higher digital literacy and resilience levels than women. In addition, girls outperformed boys in seeking help, proactive learning, and self-recovery. So, our findings revealed that girls show higher potential for self-recovery in times of digital stress, digital difficulty, and digital risk. Girls may have more protective resources related to resilience; for instance, girls tend to have better companionship, greater family cohesion, and better verbal communication skills. Girls are also willing to use more emotionally focused strategies, such as proactive help-seeking and empathy. These social and emotional resources may help girls better cope with stress, hardship, and risk, promoting their resilience.

In addition, studies show that high school students have better digital resilience than middle school students. Among them, high school sophomores performed the best in digital resilience. In other words, high school students are older than middle school students, resulting in higher levels of digital resilience among high school students. For instance, Student J described the relationship between time spent on the Internet and exposure to online risks and difficulties while arguing that people who spend more time online are more adaptable to the online environment. The findings of the present study can appropriately be manifested through the argument provided by some scholars (41) in the literature that the longer the young digital natives have been online and the more they know about cybersecurity risks, the more digitally literate they are and the more digitally sensitive they are. As a result, they will also show better resilience in the online world.

Meanwhile, adolescents from urban areas are more digitally resilient and perform better in knowing the risks, seeking help, proactive learning, and self-recovery than those from rural areas. The findings essentially replicated those reported previously based on the perspective of the program participants. For instance, Budak et al. (20) concluded that urban residents may exhibit higher levels of resilience because they can access technology more easily and quickly. For instance, Student H stated, *“This is because the network infrastructure in rural areas is underbuilt, network resources are scarce, and network fees are still expensive.”* From this, it can be seen that there are significant differences in the delivery and use of digital technologies in rural areas compared to urban areas. This can be seen in access to different technologies, IT infrastructure, or IT education. This leads to people living in rural areas being less able to adapt to the digital world than urban residents.

Adolescents from non-one-child homes outperformed adolescents from one-child families regarding digital resilience and the dimensions of knowing the dangers, requesting help, proactive learning, and self-recovery. Individuals with siblings, for example, adapt better and display better resilience online, according to student E. This is because they have more experience with peer cooperation and interaction. Only children, on the other hand, may lack these advantages, which may hurt their resilience. The interview suggests that sibling support and companionship play an essential role in the digital resilience of adolescents.

## 6 Conclusion

In modern society, the Internet is at the forefront of social development, and it plays an increasingly important role in adolescents’ daily lives, as well as bringing convenience and diversity to their lifestyles. Adolescents can communicate conveniently with their teachers and classmates through the Internet, extending the classroom outside the classroom. It enhances the impact of learning and contributes to their academic development. However, the colorful network is a sharp, double-edged sword; it makes people’s lives more convenient and rich and hides countless dangers. For example, the Internet makes many adolescents indulge in the virtual world of the network, detached from reality, but it also makes some adolescents desert their studies. These characteristics of the virtual world mean that many adolescents would rather indulge in the unreal environment all day long than face real life. Cultivating the digital resilience of adolescents to accurately recognize and scientifically respond to digital risks, challenges, and dangers will help them face the many digital challenges with ease and turn crises into opportunities. In this study, the adolescent digital resilience assessment framework was first based on relevant studies, and the Adolescent Digital Resilience Scale was designed with high reliability and validity. The study further surveyed the current situation of digital resilience among Chinese adolescents (mainly including middle school and high school students), explored the current level of digital resilience development among adolescents and the influencing factors of the individuals concerned, and provided an effective tool and data support for the scientific knowledge of the current level of digital resilience development among adolescents.

Based on the study results, three recommendations for stimulating, preserving, and empowering digital resilience in adolescents are presented. First, strategies for dealing with digital crises and dangers should be included in educational curricula to increase adolescents’ digital literacy and well-being, with educators serving as guides and supporters. Adolescents should learn to use digital tools appropriately and create digital resilience by including mental health, psychological resilience, and critical occurrences in the curriculum. Teachers should provide adequate emotional support, mindful comfort, and guidance to adolescents who encounter online dangers and obstacles in order to help them improve their sense of self-efficacy and resilience. Research has shown that teachers’ emotional support has a significant positive effect on the development of adolescents’ resilience (42), and adolescents who perceive more teacher support are better able to cope with online risks and crises (43). Second, young people’s digital interests, digital skills, and “digital optimism” can enhance adolescents’ digital resilience (44). Adolescents who believe that the Internet and digital technologies benefit social and personal development and those who have accumulated additional skills using digital technologies are more likely to be resilient self-regulators and strong self-adjusters online. This suggests that fostering essential digital competencies in young people positively impacts fostering resilience and active participation in many online environments. Therefore, in-home and school education, parents and teachers should help young people develop positive digital interests and optimistic digital attitudes and improve their digital competence and literacy, thus, their digital resilience. Third, parental attitudes and digital competence shape most adolescents’ primary caregivers, first educators, and online supervisors. Parental support, facilitation, and encouragement of adolescents’ Internet use and modeling of how to access the Internet safely are critical factors in developing adolescents’ digital resilience (45). Conversely, when parents restrict their children’s digital devices or Internet use or monitor their online activities without positive encouragement or support, they may inadvertently diminish their children’s ability to build digital resilience. In summary, assuming that building digital resilience is a complex and dynamic process, further research may examine which topics or resources positively impact adolescent digital resilience.

## 7 Implication

This study has some limitations. This study has several limitations. First, this study relied entirely on self-reported data from adolescents. Self-reporting is commonly acknowledged as a data collection strategy that might lead to data bias. A future study may consider data from other sources, such as reports from teachers, parents, and peers, as complementary information to assess adolescents’ levels of digital resilience. Second, this study primarily included middle- and high-school adolescents who are only about to complete part of the nascent stage. In addition, this study only investigated the current status of digital resilience and individual influencing factors among some Chinese adolescents. Therefore, the findings are only applicable to a portion of Chinese adolescents. Finally, in the resilience literature, digital resilience is a relatively new characteristic that requires additional manipulation and measurement.

## Data availability statement

The original contributions presented in the study are included in the article/supplementary material. Further inquiries can be directed to the corresponding author.

## Ethics statement

The studies involving humans were approved by college of education Jiangxi Normal University. The studies were conducted in accordance with the local legislation and institutional requirements. Written informed consent for participation in this study was provided by the participants’ legal guardians/next of kin. Written informed consent was obtained from the individual(s), and minor(s)’ legal guardian/next of kin, for the publication of any potentially identifiable images or data included in this article.

## Author contributions

CQ: Data curation, Methodology, Writing – original draft. NY: Writing – review & editing, Supervision.
